# A Super‐Stretchable Liquid Metal Foamed Elastomer for Tunable Control of Electromagnetic Waves and Thermal Transport

**DOI:** 10.1002/advs.202000177

**Published:** 2020-04-30

**Authors:** Dehai Yu, Yue Liao, Yingchao Song, Shilong Wang, Haoyu Wan, Yanhong Zeng, Tao Yin, Wenhao Yang, Zhizhu He

**Affiliations:** ^1^ Department of Vehicle Engineering College of Engineering China Agricultural University Beijing 100083 China

**Keywords:** elastomer composites, electromagnetic interference shielding, foamed elastomers, liquid metals, thermal conductivity switch

## Abstract

It is remarkably desirable and challenging to design a stretchable conductive material with tunable electromagnetic‐interference (EMI) shielding and heat transfer for applications in flexible electronics. However, the existing materials sustained a severe attenuation of performances when largely stretched. Here, a super‐stretchable (800% strain) liquid metal foamed elastomer composite (LMF‐EC) is reported, achieving super‐high electrical (≈10^4^ S cm^−1^) and thermal (17.6 W mK^−1^) conductivities under a large strain of 400%, which also exhibits unexpected stretching‐enhanced EMI shielding effectiveness of 85 dB due to the conductive network elongation and reorientation. By varying the liquid and solid states of LMF, the stretching can enable a multifunctional reversible switch that simultaneously regulates the thermal, electrical, and electromagnetic wave transport. Novel flexible temperature control and a thermoelectric system based on LMF‐EC is furthermore developed. This work is a significant step toward the development of smart electromagnetic and thermal regulator for stretchable electronics.

## Graphical abstract

This work reports a super‐stretchable liquid metal foamed elastomer composite with both high electrical and thermal conductivities, and electromagnetic interference shielding effectiveness of 85 dB even when strained at 400% due to the stretching enabled conductive network elongation and reorientation, which also exhibits unique multifunctional reversible switch responses for application to tunable control of electromagnetic waves and thermal transport.

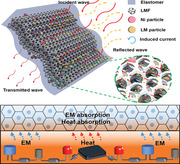

With the rapid development of flexible electronics^[^ ^1^ ^]^ toward miniaturization and high‐power densities, waste heat and electromagnetic interference (EMI) as inevitable byproducts, poses a significant influence on the electronic apparatus and even leads to degradation and malfunctioning of electronics particularly for that operating at high frequencies. Electromagnetic pollution also leads to a considerable threat to the surrounding environment and human health. EMI shielding material can weaken or eliminate adverse impacts of the spurious emissions between adjacent electronics while blocking the electromagnetic communication and heat dissipation of electronics devices. It is thus, remarkably desirable to design a smart material for tunable control of EMI shielding and temperature of flexible electronics, especially if it can automatically adapt to a large deformation without performance degradation.

There are existing two main strategies to prepare the stretchable EMI shielding material, namely developing conductive porous structure often packaged with elastic matrix, such as 2D transition metal carbides (MXene),^[^ ^2^, ^3^ ^]^ carbon nanotube (CNT)^[^ ^4^ ^]^/graphene^[^ ^5^, ^6^ ^]^/reduced graphene oxide^[^ ^7^ ^]^ foam, and carbon fiber fabric,^[^ ^8^ ^]^ or incorporating conductive fillers into elastomer composites (EC), which include CNT,^[^ ^9^, ^10^ ^]^ graphene,^[^ ^11^ ^]^ and metal particles.^[^ ^12^, ^13^ ^]^ The former can achieve a superhigh EMI shielding effectiveness (even higher than 90 dB^[^ ^2^ ^]^), but has a limited stretchable capacity (strain < 50%) due to the intrinsic rigid porous structure and was easily fractured in structure for a high strain > 200%. The latter could withstand a considerable stretching (such as a maximum strain of 232% and EMI SE of 59.8 dB under free stress for CNT@EC,^[^ ^14^ ^]^) while it also exhibits serious attenuation of EMI SE when strained due to the stretching‐induced separation between the solid conductive fillers and the decrease of electrical conduction (such as the EMI SE decrease from 45 to 35 dB at only 50% strain for silver nanowire @ EC^[^ ^12^ ^]^). Thus, the existing EMI shielding materials are hard to be adapted to stretchable electronics when sustained a great stretching.

The thermal conductivity of EMI shielding materials has a remarkable impact on the thermal dissipation of the electronic device, especially with high‐power density. However, there is a significant challenge to achieve high thermal conductivity for high‐stretchable materials,^[^ ^15^, ^16^ ^]^ which is similar to the mismatch between the electrical conduction and the stretchable capacity of the elastomer. It is desirable for many applications to the reversible tuning of the thermal conductivity, which helps to enhance the heat dissipation of electronics with a high value, while its low value could provide thermal protection against external heat shock. The available thermal conductivity switches are mainly originated from the bulk thermal conductivity change after solid/liquid or solid/solid phase transitions,^[^ ^17^ ^]^ which include the suspension,^[^ ^18^ ^]^ polymers,^[^ ^19^ ^]^ and structured materials.^[^ ^20^ ^]^ However, they are hard to achieve a high thermal conductivity contrast ratio (<10 reported in the previous experiments^[^ ^17^ ^]^) and have a low electrical conductivity so that they are not suitable for application to EMI shielding.

Chemically stable and nontoxic LM (including Gallium and BiInSn alloys with melting points near room temerature^[^ ^21^ ^]^) have attracted great interest^[^ ^22^ ^]^ and potential applications owing to high thermal/electrical conductivities and deformability, such as flexible sensors^[^ ^23^ ^]^ and electronics,^[^ ^24^ ^]^ and soft robotics.^[^ ^25^ ^]^ LM‐filled EC (LMEC)^[^ ^26^ ^]^ exhibit unique coupling of electrical,^[^ ^27^, ^28^ ^]^ thermal,^[^ ^15^, ^16^, ^29^ ^]^ and mechanical properties,^[^ ^30^, ^31^, ^32^ ^]^ such as for LM volume ratio of *ϕ* = 50% a high electrical conductivity of *σ* = 1.1 × 10^2^ S cm^−1^ though mechanical‐sintering method but with a limited strain (133%) to failure.^[^ ^33^ ^]^ Besides, LM‐filled EC foam (LM‐ECF) were developed for application in flexible electronics^[^ ^34^ ^]^ and soft robotics,^[^ ^30^ ^]^ while with a limited stretching ability (strain < 50% at room temperature). It is also noteworthy that the ECF filled with LM through immersing it into LM bath is hard to be coated tightly with EC due to the separation by LM.

In this work, we reported a super‐stretchable (800% strain) LM‐foamed EC (LMF‐EC) consisted of a porous LM structure coated with the elastic composite of the silicon matrix. The 3D conductive network of LMF constrained in the EC matrix was elongated and reorientated in the stretching direction to maintain its complete topological structure, and achieve outstanding stretching‐enhanced electrical, thermal, and EMI shielding performances. It yet exhibited superhigh electrical (≈10^4^ S cm^−1^) and thermal (17.6 W mK^−1^, ≈90 times than the pure silicon matrix) conductivities, as well as EMI SE of 85 dB under a considerable strain of 400%. A novel strategy based on the stretching combined with liquid–solid phase change of LMF was proposed to enable a multifunctional simultaneous reversible switch, such as the resistivity change ratio ≈10^9^ times, ≈16 for thermal conductivity variation (exceed most the available experiment results <10) and ≈20 for EMI SE change (reported here for the first time) were obtained, respectively. With these findings, the present work opens a highly feasible way to develop a smart multifunctional regulator for applications in stretchable electronics, flexible sensors, soft robotics, heat transfer enhancement, and energy utilization.

**Figure** **1**a is a schematic of the LMF‐EC preparation process through a cost‐effective sugar sacrificial template method. Briefly, sugar particles with grain size between 100–150 µm were tightly stacked as templates, which were then fully immersed in the Ga LM (melting point of 29.7 °C) to allow it permeate into the gap through vacuuming at 70 °C. It was then polished to make the exposed sugar be completely dissolved and washed away in an ultrasonic cold‐water bath (10 °C). The size and shape of LMF could be adjusted through the designed sugar template. The microstructure of LMF is presented in Figure 1b (also see 3D structure by micro‐CT in Movie S1, Supporting information), indicating the LM skeleton connection to form the 3D conductive network. The LMF‐EC was then prepared through immersing LMF into the uncured Pt‐catalyzed silicone (Ecoflex 00–30, Smooth‐On) elastomer composite (EC) with a coating thickness about 0.1–0.5 mm, which was then cured at the room temperature for 5 h. The LM of pure Ga was first adopted due to its slightly higher melting point (29.7 °C) than the room temperature, which results in a simple preparation process without needing a low‐temperature environment compared with that for GaIn‐based LMF (15.7 °C)^[^ ^21^ ^]^ preparation. It is noteworthy that Ga is easily melted by human body temperature (see Movie S2, Supporting Information), and still keep liquid state at room temperature (15–25 °C) for a long time (>30 days) due to its large supercooling degree (the observed solidification point below −20 °C through differential scanning calorimetry (DSC) method, see Figure S1, Supporting Information). The prepared LMF have a typical porosity of 75% to make LMF‐EC (the LM volume fraction with *ϕ* = 25%) like an elastomer (density of 2.2 g cm^−3^) with a maximum strain of 800%, which exhibit a quite contrary structure to LM‐ECF (4.7 g cm^−3^ for the same porosity of 75%) with a limited stretchable capacity (<50% strain at room temperature).^[^ ^30^, ^34^ ^]^ The super stretchability of LMF‐EC was mainly attributed to the coupling of the internal connected LM skeleton and the volume‐dominant elastomer matrix with both excellent deformability. It is found from SEM in Figure 1b that the LM skeleton was elongated and reorientated along with the coated elastic matrix to maintain its complete topological conductive network (also clearly presented in Figure S2 and Movie S3, Supporting Information), which are crucial to enable both high electrical and thermal conduction under large stretching.

**Figure 1:**
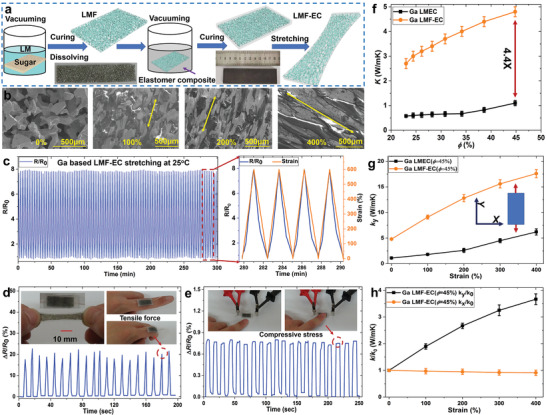
Schematic of LMF‐EC preparation and thermal‐electrical properties characterization. a) Illustration of LMF‐EC (Ga) preparation process. b) SEM images of stretched Ga‐based LMF‐EC with strains of 0%, 100%, 200%, and 400%, respectively. c) The resistance changes of Ga‐based LMF‐EC (3 mm × 13 mm × 30 mm) under cyclic stretching loading at room temperature. The relative resistance changes of Ga‐based LMF‐EC with size (1 mm × 13 mm × 25 mm) for monitoring d) finger bending and e) touching. Photograph of LMF‐EC and its elongation through cold‐stretching and hot‐stretching. f) Effective thermal conductivity of Ga‐based LMF‐EC and LMEC versus *ϕ*. g) The thermal conductivity of Ga‐based LMF‐EC (*ϕ* = 45%) under stretching at room temperature. h) The stretching‐enabled directional dependence of *K* for Ga‐based LMF‐EC (*ϕ* = 45%).

The LMF‐EC exhibit a superhigh volumetric electrical conductivity of *σ* = 1.8 × 10^3^ S cm^−1^ under free strain for a small *ϕ* = 25%, and a remarkable increase to *σ* = 1.1 × 10^4^ S cm^−1^ when strained to 600%, respectively. The electric resistance of conductive elastomer without deformation was estimated by *R*_0_ = *L*_0_/*A*_0_/*σ*_0_, where *L*_0_ is its length, *A*_0_ denotes the cross‐section area, and *σ*_0_ for the effective electrical conductivity, respectively. For incompressible conductive elastomer, the effective electrical conductivity of conductive elastomer under stretching was estimated^[^ ^36^ ^]^ by *σ*/*σ*_0_ = (1+ *ɛ*)^2^ *R*_0_/*R*, where the strain of *ɛ* = (*L*−*L*_0_)/*L*_0_ was defined. As shown in Figure 1c, the resistance change ratio *R*/*R*_0_ of LMF‐EC presents excellent cycling durability of reversibly linear resistance–strain correlation. It is completely different from the pure LM conductor^[^ ^31^ ^]^ with a second‐power relation between *R*/*R*_0_ and *ɛ*. The applied force (such as stretching, bending, and compression) could induce the LMF skeleton deformation to change its electric resistance, which could be used as the force sensor, such as application for monitoring finger bending and touching (see Figure 1d,e). The LMF‐EC also achieves a stretching‐enhanced thermal conductivity when strained. The thermal conductivity of LMF with direction‐independent porous structure could be roughly estimated by a simplified model^[^ ^35^ ^]^ of *K* = *K*_LM_ *ϕ*/3 for small *ϕ*. The estimated *K* = 2.8 W mK^−1^ is consistent with the measurement result of 2.7 ± 0.2 W mK^−1^ for LMF (*K*_Ga_ = 33.2 W mK^−1^, *ϕ* = 25%). The heat conduction of LMF‐EC could be significantly improved through adding the LM microparticles (10–20 µm, see Experimental Section for material fabrication) into the EC matrix, where the total LM volume fraction of *ϕ* = 25% + *ϕ*_LMP_ = 25–45% was considered. The thermal conductivity of LMF‐EC is remarkably higher than that of LMEC with the same *ϕ* as shown in Figure 1f, such as a high *K* = 4.8 ± 0.2 W mK^−1^ of LMF‐EC is about 4.4 times than that (*K* = 1.1 ± 0.1 W mK^−1^) of LMEC with *ϕ* = 45%. The stretching‐enhanced thermal conductivities were observed for both LMF‐EC and LMEC, as presented in Figure 1g, and a unique directional dependence of heat conduction was also obtained (see Figure 1f). However, LMF‐EC would achieve a remarkably larger *K* than that of LMEC with the same *ϕ*, such as for *ϕ* = 45%, *K*_y_ = 17.6 ± 0.8 W mK^−1^ along stretching direction for LMF‐EC is 2.8 times than that (*K*_y_ = 6.2 ± 0.6 W mK^−1^) for LMEC under 400% strain, and ≈90 times than pure silicon matrix (Ecoflex 00–30, 0.2 W mK^−1^), respectively. More importantly, LMF‐EC exhibit superhigh electrical conduction under large stretching to be significant desirable for stretchable electronics, while LMEC without additional pretreatment is insulating.^[^ ^16^ ^]^ The stretching‐enhanced electrical and thermal conduction of LMF‐EC could be attributed to the 3D network of LMF with super high deformation capacity, which was elongated and reorientated along with the elastic matrix to maintain its complete topological structure, and enabled an unexpected conduction enhancement. It is theoretically explained in Supporting Information text, and demonstrated by experiment results.

The super‐stretchable LMF‐EC holds excellent promise for EMI shielding due to its 3D excellent electrically conductive network. The existing stretchable EMI shielding materials were often composited of the elastic matrix filled with the solid conductive particles (such as CNT and metals),^[^ ^9^ ^]^ which suffer from a large decrease of EMI SE when strained.^[^ ^12^ ^]^ It is about 10 dB at X band (8.2–14.5 GHz) for the LMEC (filled with *ϕ* = 25% solid BiInSn microparticles) strip with a thickness of 3.6 mm under free stress, while it approaches zero under 400% strain at room temperature (see **Figure** **2**a). It is interesting to find that 400% strain could achieve a 150% increase of EMI SE (25 dB, see Figure 2b) for LMEC (filled with *ϕ* = 25% Ga droplets). The solid particles and droplets would enable LMEC with opposite EMI shielding behavior when strained, which is because the former would separate from each other under strain to cause electrical conduction drop drastically, while deformation of the latter along with the elastomer matrix as illustrated in Figure 2c (also observed in Figure S3, Supporting Information) to maintain its conductance. It is surprising to find that the stretching could enable LMF‐EC (*ϕ* = 25%) to achieve a remarkable increase from 57 dB under free stress to 85 dB at 400% strain, as shown in Figure 2d, which is significantly higher than that of LMEC with the same *ϕ `*(see Figure 2e). It is also noteworthy that a 400% strain would lead to the thickness reduction of the LMF‐EC from 3.6 to 2 mm. For most existing EMI shielding materials, however, the thickness reduction would weaken the EMI shielding performance. This unique strategy of stretching‐enhanced superhigh EMI SE has not yet been reported in the literature. The total shielding efficiency (SET) of LMF‐EC mainly includes contributions from the absorption (SEA) and reflection (SER) and the multiple reflections (often ignored for SET > 15 dB), respectively. To avoid the second EM pollution, it is advisable to achieve high SEA. As shown in Figure 2f, adding the LM droplets (or microparticles of Ni) into EC matrix would improve SEA fraction from 60% for LMF‐EC (*ϕ* = 25%) to 69% for LMF‐EC (*ϕ* = 40% + 5%Ni) under stress‐free, respectively, which exhibits a considerably larger absorption and lower reflection due to providing more interfaces for multiple reflection and scattering of electromagnetic waves. The introduction of Ni microparticles into the elastic matrix could enhance the magnetic losses to improve the performance of EMI shielding absorption. Figure 2g displays the comparison between LMF‐EC and other EMI shielding materials, and indicate that only EMI‐EC could still preserve high EMI SE (>80 dB) under a deep stretching (strain = 400%), which is critical for applications to stretchable electronics.

**Figure 2:**
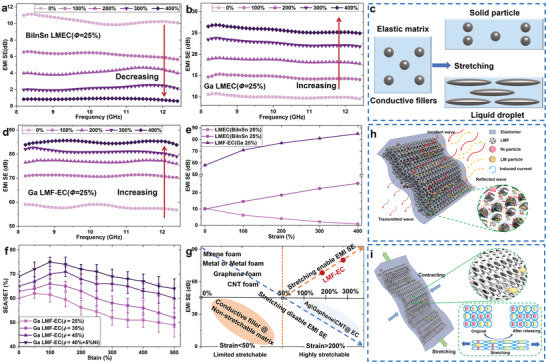
Thermal‐mechanically enabled EMI shielding performance. a) Stretching disabled EMI SE of BiInSn‐based LMEC (*ϕ* = 25%) at room temperature. b) Stretching enabled EMI SE of Ga‐based LMEC (*ϕ* = 25%) at room temperature. c) Illustration of the solid particles and liquid droplets in elastomer matrix response to the stretching. d) Stretching‐enabled EMI SE of Ga‐based LMF‐EC (*ϕ* = 25%) at room temperature. e) The comparison of EMI SE for BiInSn‐based and Ga‐based LMEC, and Ga‐based LMF‐EC with the same *ϕ* = 25% when strained. f) The fraction of SEA versus the stretching for Ga‐based LMF‐EC when strained. g) The comparison between LMF‐EC with the other EMI shielding materials. Illustrations of EMI shielding mechanism of LMF‐EC under h) free stress and i) stretching.

The high EMI shielding performance of LMF‐EC mainly originates from its internal excellent conductive network, as illustrated in Figure 2h. When electromagnetic wave encounters the curved skeleton surface of the LMF, the scattering of the incident wave occurs and dramatically reduces the intensity of the reflected wave in the opposite direction of the incident wave, which is in marked contrast to a flat surface of pure metal that makes electromagnetic wave directly reflected into the air. The connected opened‐foam structure can attenuate the incident electromagnetic waves by scattering, reflecting, and absorbing between the metallic skeleton and the filled LM particles. Besides, the multi‐layered foams would induce multiple reflections and absorptions of electromagnetic waves due to the impedance mismatch between the layers, which make them stranded inside the LMF‐EC before being absorbed and transformed into heat. It is noteworthy that the electrical conductivity is the primary factor determining SEA and SER, which led to $\mathrm{SET}\;=\;\left[39.5\;+\;10\log\frac{\sigma }{2\pi f\mu }\right]\;+\;8.7d\sqrt{\pi f\mu \sigma }$, where *d* adopted the thickness of the monolayer LMF and is not the total thickness of LMF‐EC, *f* and *μ* are frequency and permeability,^[^ ^6^, ^7^ ^]^ respectively. The predicted SET = 63 dB for LMF‐EC (*ϕ* = 25%) is consistent with our experiment result of 57 dB. During stretching, as illustrated in Figure 2i, the liquid skeleton of the LMF was deformed along with the elastomer matrix, while the corresponding vertical cross‐sections contracted and led to a sharp reduction of layer interval between the foams. Consequently, the porosity of LMF would decrease in the contracted cross‐sections to enhance EMI performance. Also, the stretching could significantly increase the effective electrical conductivity, which helps to improve the EMI shielding.

The reversible liquid–solid phase transition of LMF would enable LMF‐EC to exhibit variable stiffness and shape‐memory behavior. It is more convenient to dynamically control phase transition of BiInSn‐based (melting temperature of 60.2 °C) LMF through simple heating and cooling at room temperature compared with that of Ga due to its large supercooling degree. As shown in Figure **3**a, LMF‐EC (BiInSn) exhibits excellent stretchable performance for both solid and liquid LMF, which achieved a strain of 600% for solid LMF (cold‐stretching at 25 °C) and 700% for liquid LMF (hot‐stretching at 70 °C), respectively. The stress–strain curves of LMF‐EC (BiInSn, *ϕ* = 25%) presented in Figure 3b have demonstrated that the strain at the break is over 800% for both solid and liquid LMF skeleton due to the super‐elastic EC matrix. The stress–strain behavior of LMF‐EC can be furthermore regulated by filling LM particles into the EC matrix. Increasing the filling ratio (volume fractions *ϕ*_LMP_ = 0–20%) of LM particles would slightly decrease the maximum strain of LMF‐EC (corresponding to *ϕ* = 25–45%) while enhances its tensile modulus (see Figure S4, Supporting Information). The LMF‐EC stiffness is easily and dynamically controlled through the cyclically cooling‐heating method and enables shape‐memory behavior. The LMF‐EC strip (3.6 mm × 13 mm × 30 mm) is load‐bearing at room temperature (see Figure 3c; Movie S4, Supporting Information), and then softens and deforms through hot‐stretching. The designed deformation is maintained when cooled to room temperature, which is attributed to the reshaped LMF skeleton as a supporting structure. The inner residual stresses of LMF‐EC make it to keep a variety of the designed shape (more examples presented in Figure S5 and Movie S5, Supporting Information), which could be quickly released only though heating above the melting point. This temperature‐enabled shape‐memory function can be enriched through filling nickel microparticles into EC, which was then driven by the magnetic force due to the nickel magnetization in an external magnetic field gradient. A conceptual demonstration of simultaneous magnetic‐thermal contactless actuation is shown in Movie S6, Supporting Information. It is interesting to find from Figure 3d (also see Movie S7, Supporting Information) that the solid skeleton as the conductive path was completely ruptured under a cold‐stretching at 20% strain to make LED array light off, and recovery lighting once heating over the melting point. The fractured LMF constrained in the EC matrix could recrystallize through a liquid–solid phase change to make conductive network reconnection. This unique self‐healing process was observed through the microscope camera as presented in Figure 3e (the corresponding dynamical process recorded in Movie S8, Supporting Information), which demonstrated that the liquid capillary force drives the fractured LM skeleton interconnection. The compression testing experiments shown in Figure 3f indicated that LMF‐EC block (10 × 20 × 20 mm) has a large stress tolerance capacity with a plastic deformation characteristic, which can bear heavy objects about 50 times its own weight (see photograph in Figure 3f).

**Figure 3:**
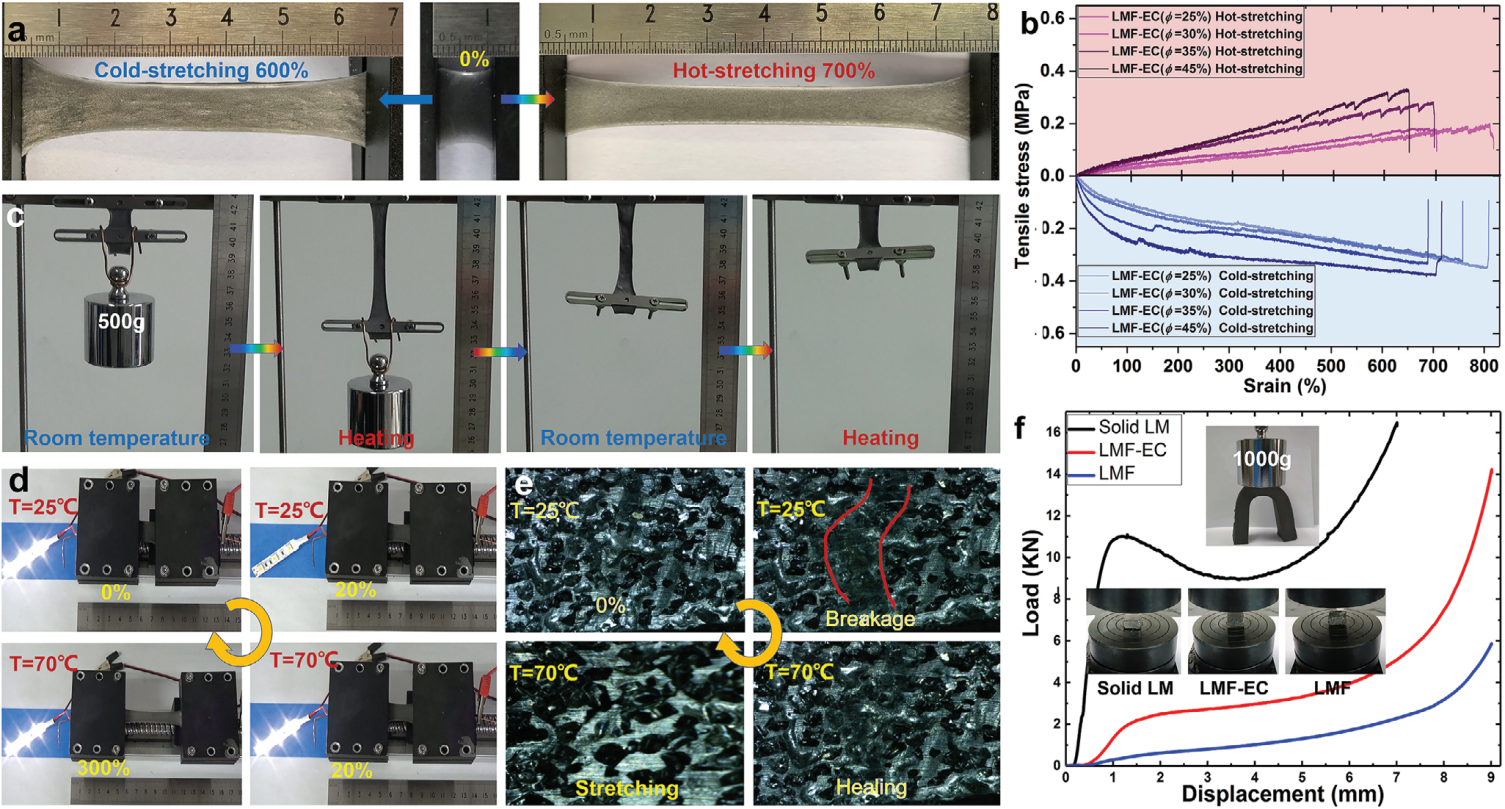
Thermal‐mechanically enabled variable stiffness and shape‐memory behavior. a) Photograph of BiInSn‐based LMF‐EC and its elongation through cold‐stretching and hot‐stretching. b) The stress–strain curves of LMF‐EC with liquid and solid skeletons. c) BiInSn‐based LMF‐EC loaded with a 500 g load in solid‐state, softened and deformed under the load. Stretching enabled EMI SE of Ga‐based LMEC (*ϕ* = 25%) at room temperature. d) Photographs of the stretchable BiInSn‐based LMF‐EC as the electrical interconnect for LED lighting control through its thermal‐mechanically enabled transition of insulator–conductor. e) Photos of BiInSn skeleton fractures formation when strained at 25 °C and self‐heal at 70 °C. f) BiInSn‐based LMF‐EC loaded with a 500 g load in solid‐state, softened and deformed under the load.

The stretching, coupled with a reversible liquid–solid phase transition of LM, would trigger LMF‐EC to present unique tunable thermal, electrical, and EMI shielding switches due to the different structure change of LMF induced by cold and hot stretching. It is found from DSC curves in **Figure** **4**a that the melting (60.2 °C) and freezing (57.4 °C) points of BiInSn‐based LMF approach closely and allow to control its solid–liquid transition reversibly, which is demonstrated in Figure 4b. LMF‐EC has a large latent heat of 117 MJ m^−3^ for *ϕ* = 50% (see Figure 4a and Figure S6, Supporting Information), and can be used as a passive heat sink for flexible electronics without additional power input, which presents almost constant temperature platform during reversible heat charge and discharge processes (see Figure 4b). The thermal conductivity of BiInSn‐based LMF‐EC has similar behavior with that of Ga‐based LMF‐EC under free stress, as presented in Figure 4c. However, BiInSn‐based LMF‐EC (*ϕ* = 45%, *K*_BiInSn_ = 19.2 W mK^−1^) could increase from 2.8 ± 0.1 W mK^−1^ at free strain to 8.1 ± 0.3 W mK^−1^ through pretreatment of hot‐stretching at 400% strain, while decrease to 0.5 ± 0.2 W mK^−1^ with a cold‐stretching with the same strain (see Figure 4d) due to the LMF fractures. It creates a high thermal conductivity contrast ratio of about 16 times under the same strain, which was higher than most of the existing experimental results^[^ ^18^, ^19^ ^]^ (<10). It was also demonstrated that this reversible high‐contrast ratio has excellent cycling durability, as presented in Figure 4e. The liquid–solid phase transition of LMF would also allow LMF‐EC as a reversible transitional insulator and conductor (TIC) through cold and hot stretching, which achieved a huge and reversible resistivity change (more than 10^9^ times), as shown in Figure 4f. Thus, LMF‐EC can be used as a smart switch, which is more convenient to implement than that through extreme‐low temperature.^[^ ^27^ ^]^ More importantly, the cold–hot stretching would enable LMF‐EC to exhibit a unique EMI shielding switch. Figure 4g shows that EMI SE of LMF‐EC (*ϕ* = 25%) continuously increased to above 80 dB (strain at 400%) after pretreatment of hot‐stretching, while decreased to 4 dB after cold‐stretching at the same strain. The cycle‐test results shown in Figure 4h demonstrated that a high EMI SE contrast ratio of about 20 was obtained through cold and hot stretching. This feature could be used to control electromagnetic waves transport tunably.

**Figure 4:**
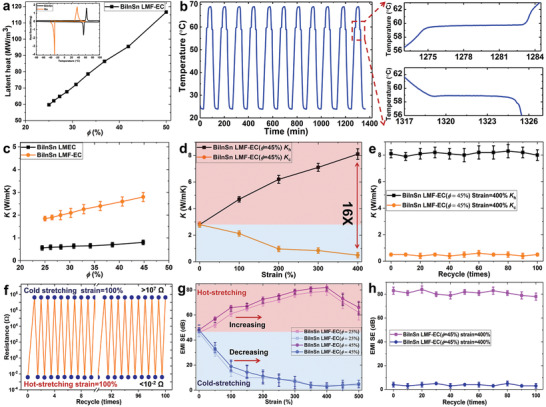
Thermal‐mechanically enabled EMI shielding performance. a) The latent heat density of BiInSn‐based LMF‐EC versus the LM volume ratio. b) Cycling durability of solid–liquid phase transition of BiInSn‐based LMF‐EC for providing constant temperature platform. c) Effective thermal conductivity of BiInSn‐based LMF‐EC and LMEC versus *ϕ*, at room temperature. d) Tunable thermal conductivity of BiInSn‐based LMF‐EC (*ϕ* = 45%) after hot‐stretching and cold‐stretching, measured at room temperature. e) Cycling durability of thermal conductivity of BiInSn‐based LMF‐EC (*ϕ* = 45%) after hot‐stretching and cold‐stretching. f) The resistance change of BiInSn‐based LMF‐EC between insulator and conductor for 100 cycles under the cold–hot stretching regulation. g) Tunable EMI SE of BiInSn‐based LMF‐EC through hot‐stretching and cold‐stretching. h) Cycling durability of EMI SE of BiInSn‐based LMF‐EC (*ϕ* = 45%) after hot‐stretching and cold‐stretching.

A simultaneously coupled EMI shielding and temperature control of flexible electronics were illustrated in **Figure** **5**a, where LMF‐EC exhibit unique heat and electromagnetic wave absorption. The flexible film with a size of 1 mm × 25 mm × 100 mm and heating power of 4W was used to simulate a deformable electronic device. The prepared LMF‐EC with the size of 8 mm × 25 mm × 100 mm is tightly contacted with the flexible film, which could inhibit the temperature rise rate of the simulated power device when the temperature reaches up to 60 °C (see Figure 5b). However, the temperature rise becomes rapid without LMF‐EC, as shown in Figure 5c. We also demonstrated that LMF‐EC as the heat sink could be easily remodeled as needed to control the temperature of the flexible electronics with complicated shape (see examples in Figure S7, Supporting Information). The LM with both high electrical and thermal conductivities has been applied for flexible thermoelectric generator (TEG).^[^ ^36^ ^]^ Here, we demonstrated that the application of LMF‐EC for TEG was based on its high latent heat density. As illustrated in Figure 5d, the periodically heated LMF‐EC (20 mm × 40 mm × 40 mm) is tightly attached to the hot side of the commercial TEG module (TEG1‐142, 3.2 mm × 40 mm × 40 mm) for cycling heat discharging and electric power generation. It is observed from Figure 5e that both output voltage and current keep a nearly constant platform during solid–liquid phase transition due to the little change of temperature difference (Δ*T*) between the hot and cold sides of TEG (see Figure 5f). The TEG module can obtain an output power of 4–6 mW through harvesting the thermal energy of LMF‐EC, which is high enough to power many low‐power sensors. The main advantage of the developed LMF‐EC used for the TEG module is that it could be easily remodeled to enhance the heat transfer efficiency for both charging and discharging. Besides, the thermal energy could be held on the LMF‐EC through latent heat in advance, which then releases heat to the TEG module when needed.

**Figure 5:**
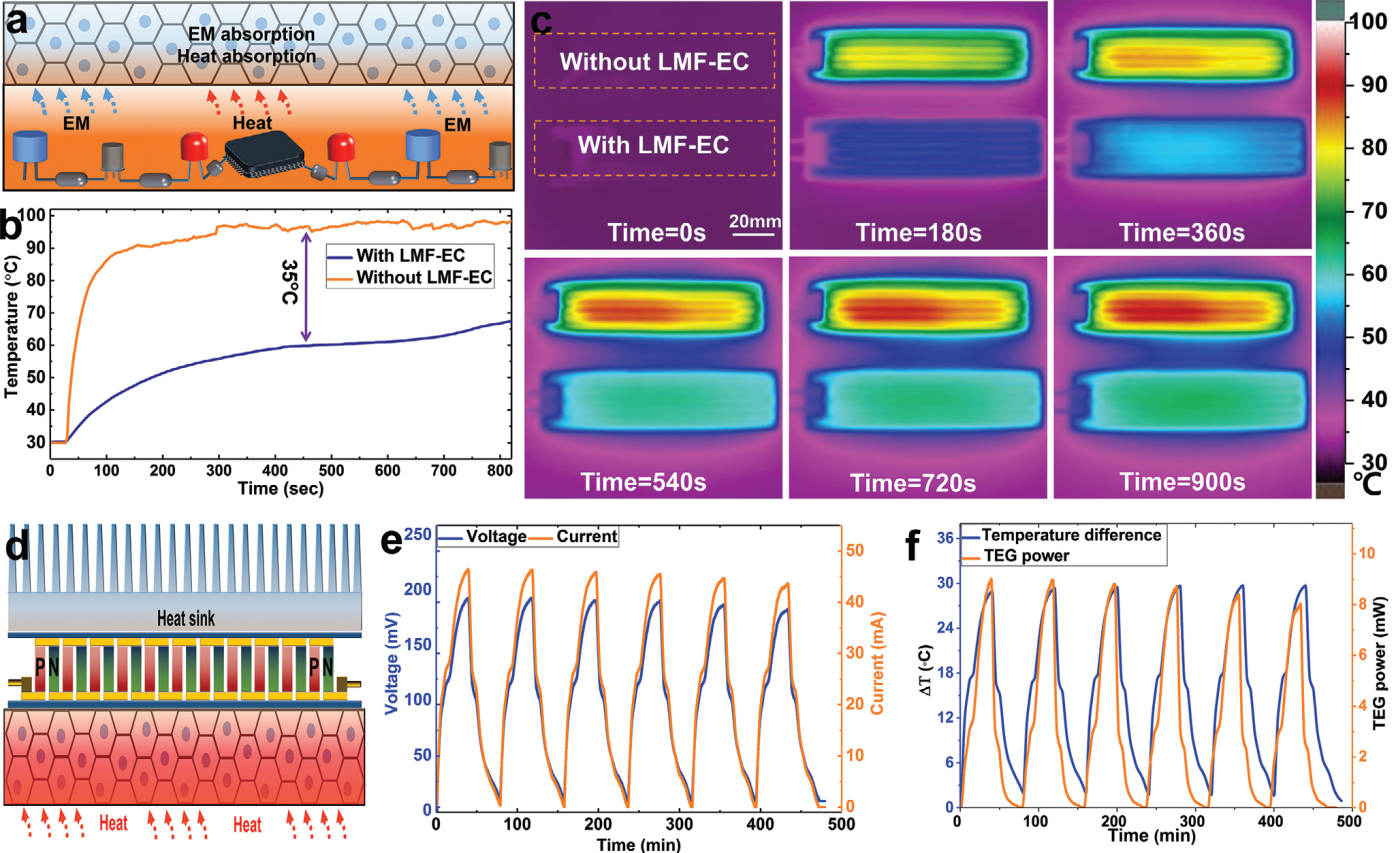
Thermal control and conversion based on LMF‐EC. a) Illustration of simultaneously coupled EMI shielding and temperature control of flexible electronics. b) The surface temperature of the flexible film with and without LMF‐EC. c) Thermal infrared images of the time‐dependent surface temperature evolution of the flexible film. d) Illustration of a novel flexible thermoelectric system integrated with the LMF‐EC for thermal energy harvesting. e) The output voltage and current of TEG. f) The temperature difference (Δ*T*) between the hot and cold sides of TEG and output power.

In summary, a super‐stretchable (800% strain) LMF‐EC reported here have both high electrical (≈10^4^ S cm^−1^) and thermal (17.8 W mK^−1^) conductivities, as well as EMI SE of 85 dB even when strained at 400% due to the stretching‐enabled conductive network elongation and reorientation. It also exhibits a unique simultaneously thermal‐electrical‐mechanically coupled response, the reversible TIC (resistivity changes more than 10^9^ times), thermal switch (reversible *K* changes about 16 times), and EMI shielding switch (reversible EMI SE changes about 20 times) through cold and hot stretching were demonstrated. To the best of our knowledge, almost all the previously reported elastomer composites do not have such multifunctional properties, respectively, the considerable enhancement of EMI SE by stretching was observed here for the first time. These properties of LMF‐EC would be of great benefit to stretchable electronics, such as flexible Ga‐based LMF‐EC for application in wearable electronics. For BiInSn‐based LMF‐EC (*ϕ* = 45%), however, hot‐stretching can enhance EMI shielding performance (high EMI SE of 83 dB) and heat dissipation (high *K* of 8.1 W mK^−1^ and latent heat of 117 MJ m^−3^) when electronics was stretched, while its cold‐stretching can allow electromagnetic communication (low EMI SE of 4 dB) between electronics and provide thermal protection (low *K* of 0.5 W mK^−1^) to external heat shock. The present work represented a significant step toward the development of smart electromagnetic and thermal regulator for stretchable electronics.

## Experimental Section

##### Preparation of LMF‐EC

Nickel microparticles (2–5 µm) from Beijing Deke Island Gold Technology Co., Ltd., and silicone of Ecoflex 00–30 from Smooth‐On were used. The eutectic Bi_31.6_In_48.8_Sn_19.6_ alloy (EBiInSn) is composed of 31.6 wt% bismuth, 48.8 wt% indium, and 19.6 wt% tin. The metal elements of Ga, Bi, In, and Sn were supplied by Zhuzhou Yilong Hung Industrial Co. Ltd, which was mixed by the given proportion at 200 °C in a vacuum drying oven for 4 h to prepare BiInSn. The elastomer composite (EC) was prepared through mixing BiInSn and Ni particles into parts of Ecoflex00–30 A and Ecoflex00–30 B separately using an electric mortar with 2500 rpm for 20–30 min at 70 °C. The liquid BiInSn was easily stirred to fabricate microliquid metal droplets with sizes 10–20 µm. The prepared silicone composites of parts A and B were then mixed by an electric mortar with 100 rpm for 3–5 min, which were then filled into LMF and cured in a vacuum drying oven for 5 h at 50 °C to obtain LMF‐EC. All the samples of LMF‐EC have a coating shell thickness of about 0.5–0.8 mm.

##### Characterization of LMF‐EC

SEM images were obtained using a Hitachi SU3500 SEM, a Xradia 410 Versa (Carl Zeiss) micro computed tomography (Micro‐CT) was applied to characterize the 3D microstructure of LMF, and a digital metalloscope (GMM‐550P, Shanghai Optical Instrument Factory) was also used to take the microscopy images of EMF‐EC.

##### Electrical and Mechanical Characterization

A linear motor is used to test the mechanical deformation of the LMF‐EC sample to measure its electrical characteristics by Keithley 2450 source measuring unit (SMU), where the two ends of LMF‐EC were electrically connected through copper electrodes. In the compression (WDW‐100E, Jinan Times Trial Machine Co., Ltd.) and stretching test (Instron 5567 mechanical testing machine), the block sample (10 × 20 × 20 mm) and strip sample (3.6 × 10 × 40 mm) were uniformly compressed with a speed of 1 mm min^−1^ and stretched with a speed of 10 mm min^−1^.

##### EMI Shielding Performance Measure

Keysight ENA network analyzer E5063A (100kHZ–18 GHz) was employed to analyze the EMI‐shielding performances of EMF‐EC or EC in the frequency range of 8.2–12.4 GHz based on a coaxial flange method. The test platform was shown in Figure S8, Supporting Information. All the test samples had a size of 3.6 × 80 × 60 mm.

##### Thermal Conductivity Measure

A transient hot‐wire method (THW)^[^ ^16^, ^17^ ^]^ was used to measure the thermal conductivity of LMF‐EC. The tested sample has a dimension of 80 × 60 × 5 mm. A 25 µm diameter Pt wire with a length 40 mm was inserted into the gap of the samples (illustrated in Figure S9, Supporting Information), two ends of which were soldered to copper and connected in a four‐probe setup to a Keithley 2450 SMU. The Pt wire was subjected to a current pulse (0.9 s) and measured the voltage over 50 data points. The currents between 50 to 200 mA were selected to get enough signal‐to‐noise ratio. The temperature change versus time (see Figure S9, Supporting Information) was obtained by calculating the resistances, which were then used to estimate the thermal conductivity of matrixes.^[^ ^17^ ^]^ THW was demonstrated by measuring and comparison with the known water thermal conductivity (0.6 W mK^−1^ at room temperature; see Figure S9, Supporting Information). All the *K* was measured at room temperature (about 25 °C).

##### Phase Change Measure

The solid–liquid phase change behavior of BiInSn and EC were characterized by DSC (200F3Maia, NETZSCH). Samples with the mass of 10 mg were put into aluminum pans to cool and heat at the ramp rate of 10 °C min^−1^ in the temperature range of −80 to 100 °C. The recycle tests were through a low/high‐temperature test chamber (BTH‐225C, Dongguan Baer Test Equipment Co., Ltd.) with heating and cooling rates of about 1.5 °C min^−1^ between 25–70 °C. The temperature of the sample was recorded by T‐type thermocouples through the Agilent 34970A data acquisition instrument. A Ti55FT Fluke Thermography infrared thermal camera was used to obtain the thermal image of the sample surface temperature in the experiment of thermal control based on LMF‐EC.

## Conflict of Interest

The authors declare no conflict of interest.

## Supporting information

Supporting Information

Supplemental Movie 1

Supplemental Movie 2

Supplemental Movie 3

Supplemental Movie 4

Supplemental Movie 5

Supplemental Movie 6

Supplemental Movie 7

Supplemental Movie 8
