# Supplementary material for: A Super‐Stretchable Liquid Metal Foamed Elastomer for Tunable Control of Electromagnetic Waves and Thermal Transport
Source: Adv Sci (Weinh). 2020 Apr 30;7(12):2000177. doi: 10.1002/advs.202000177 (PMC7312308; doi:10.1002/advs.202000177)
Supplement: Supplementary file 1 — Supporting Information [file ADVS-7-2000177-s001.pdf]

Copyright WILEY-VCH Verlag GmbH & Co. KGaA, 69469 Weinheim, Germany, 2020.

Supporting Information

A Super-stretchable Liquid Metal Foamed Elastomer for Tunable Control of Electromagnetic Waves and Thermal transport

*Dehai Yu, Yue Liao, Yingchao Song, Shilong Wang, Haoyu Wan, Yanhong Zeng, Tao Yin, Wenhao Yang, Zhizhu He**

Supporting Information Text

To quantitatively explain the mechanism of stretching-enabled enhancement of thermal conductivity for LMF-EC, its foam frame was assumed as the composition of the tetrakaidekahedron structure (a fourteen-sided polyhedron), which was often used to represent the foam microstructure. Open-celled unit foam is constructed of eight hexagonal faces, four vertical rhombic faces and two horizontal square faces, where the hexagonal faces have two sides of length L_1 and four sides of length L_2 (see Figure 1a). The cell height H and width D can be expressed as[1]

$$\begin{cases} H = 4L_2 \sin\theta \\ D = 2L_2 \cos\theta + \sqrt{2}L_1 \end{cases} \tag{1}$$

Thus, for the elongated foam unit, the strain along the y-direction could be given as

$$\varepsilon = \left(\frac{4\sin\theta}{2\cos\theta + \sqrt{2}\,L_1/L_2} \right)^{2/3} - 1 \tag{2}$$

For θ=45° and L_1/L_2=1, it is corresponding to the initial state ($\varepsilon = 0$). According to the results[2] of Bai et al., the thermal conductivity of foam along the stretching direction could be expressed as

$$\frac{K_y}{K_{LM}} = \frac{\phi}{1 + \frac{1}{2}L_1/L_2}\cos^2\theta \tag{3}$$

For θ=45° and L_1/L_2=1, $K_0 = \phi K_{LM}/3$, which is corresponding to the isotropic open-celled foam. When the LMF was stretched along the y-direction, both values of L_1/L_2 and θ become small, which thus enhanced K_y. According to our experimental observation, the change of θ has a more remarkable for a smaller strain compared with the variation of L_1/L_2. For simplification of discussion, L_1/L_2=1 was assumed for a little ε. According to Equation(2) and (3), we could find the relation between K_y/K_0 and ε though numerical method, as shown in Figure 1b. The theoretical evaluation of K_y/K_0 is consistent with the experiment results. It is noteworthy that the structure of the foam would become more complicated with a more considerable stretching, which cannot be captured by Equation (2) and

(3). Besides, the thermal effect of the elastomer matrix can be omitted for Equation (3) when the pure silicon matrix was considered due to its extreme thermal conductivity (0.2W/mK). However, the elastomer composites filled with LM particles would have a significant impact on the thermal conductivity of LMF-EC, which could furthermore enhance its thermal conduction when strained.

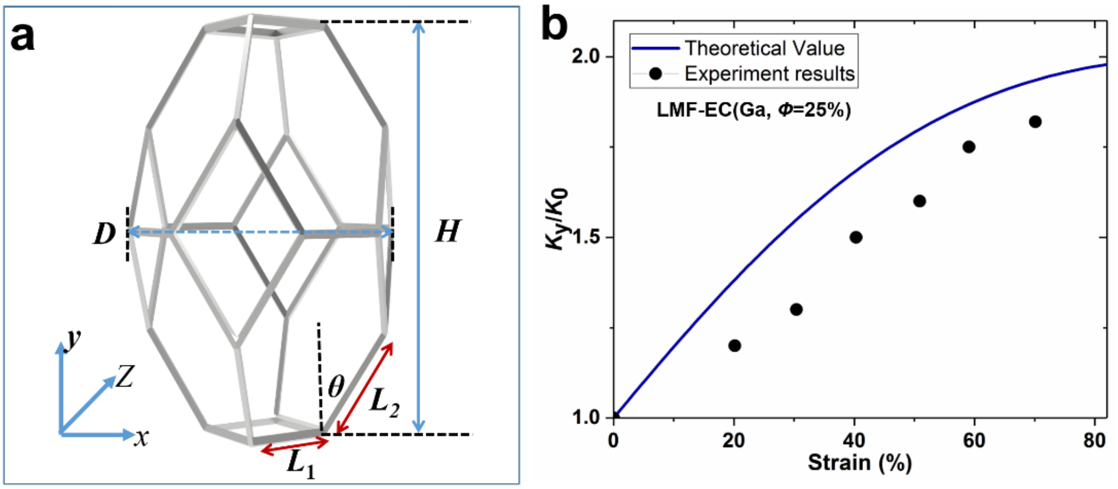

Figure 1 a) the geometry of foam microstructure, b) theoretical evaluation and experimental result of K_y/K_0 for LMF-EC(Ga, ϕ =25%) without the LM particles filled.

For the LM, the relation of the electronic and heat conductivities can be determined by the Weidemann Franz Law, k/σ=LT, where L=2.44×10^{-8} W/(S·K) is the Lorenz Number, and T is the absolute temperature. This linear relation indicates that the enhancement mechanism of thermal conductivity for LMF is suitable for its electric conductivity. The results also demonstrated that the enhancement performance for thermal conductivity is consistent with that for electric conductivity.

References

[1] R. M. Sullivan .L J. Ghosn, Int J Eng Sci, 2009,47, 990
[2] X. H. Bai, C. L. Liu, A. Nakayama, Int J Heat Mass Tran 2020, 148, 119064.

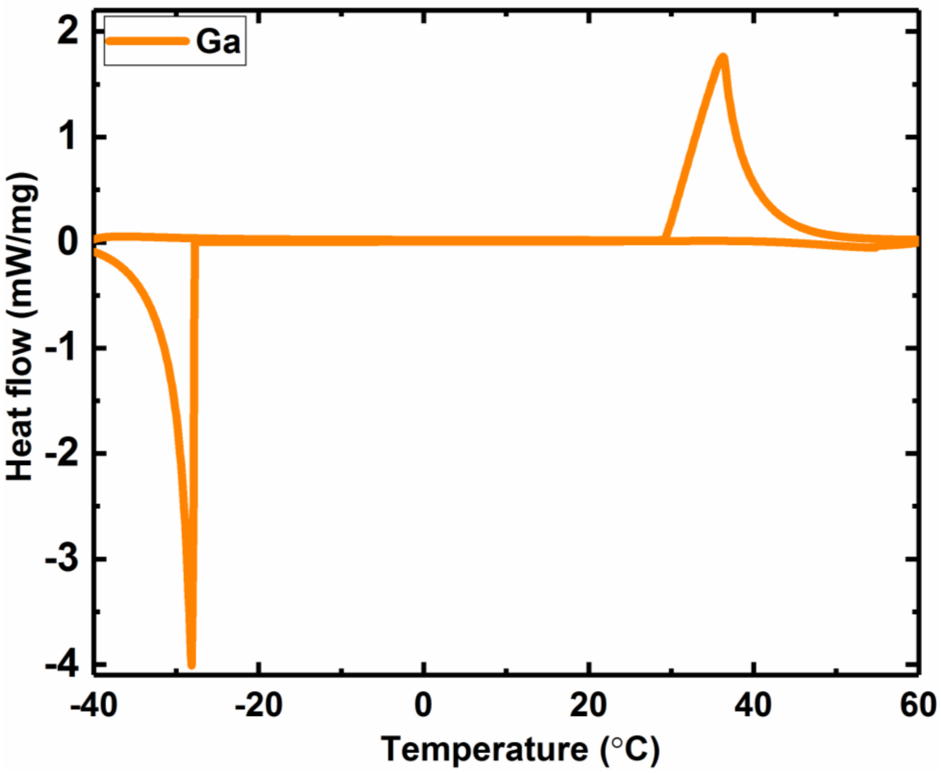

Figure S1 The DSC curves of gallium.

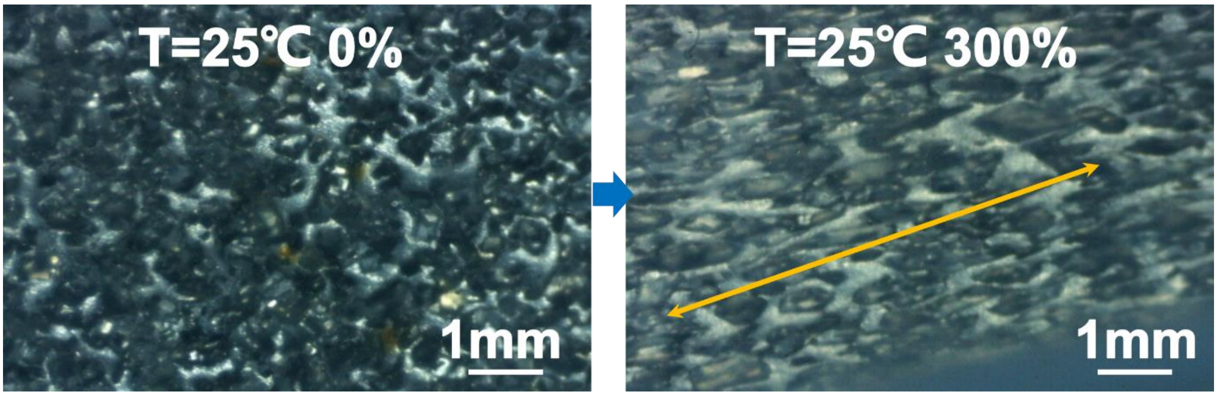

Figure S2. Optical image of the Ga-based LMF-EC with a strain of 300%.

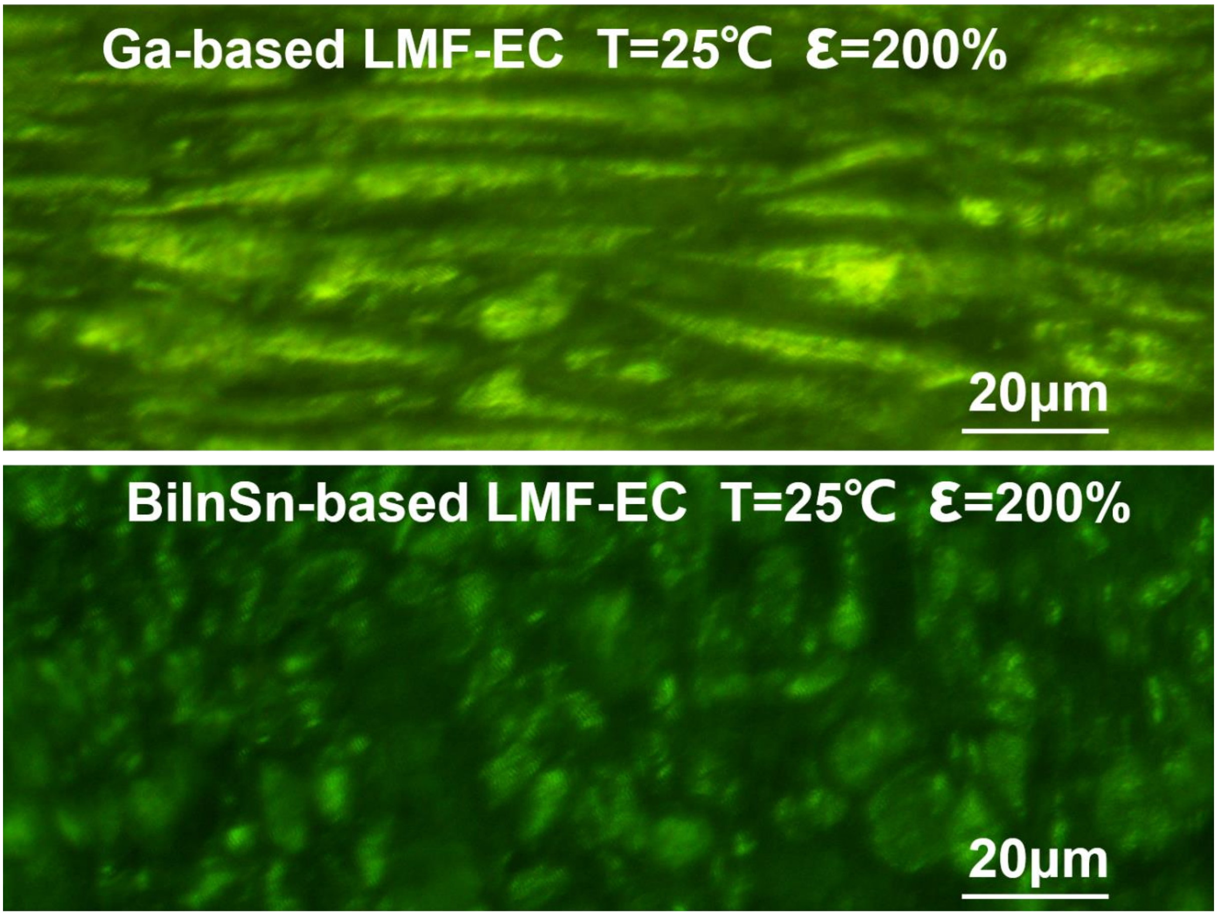

Figure S3 The different behaviors of LM microparticles in EC under stretching with 200%.

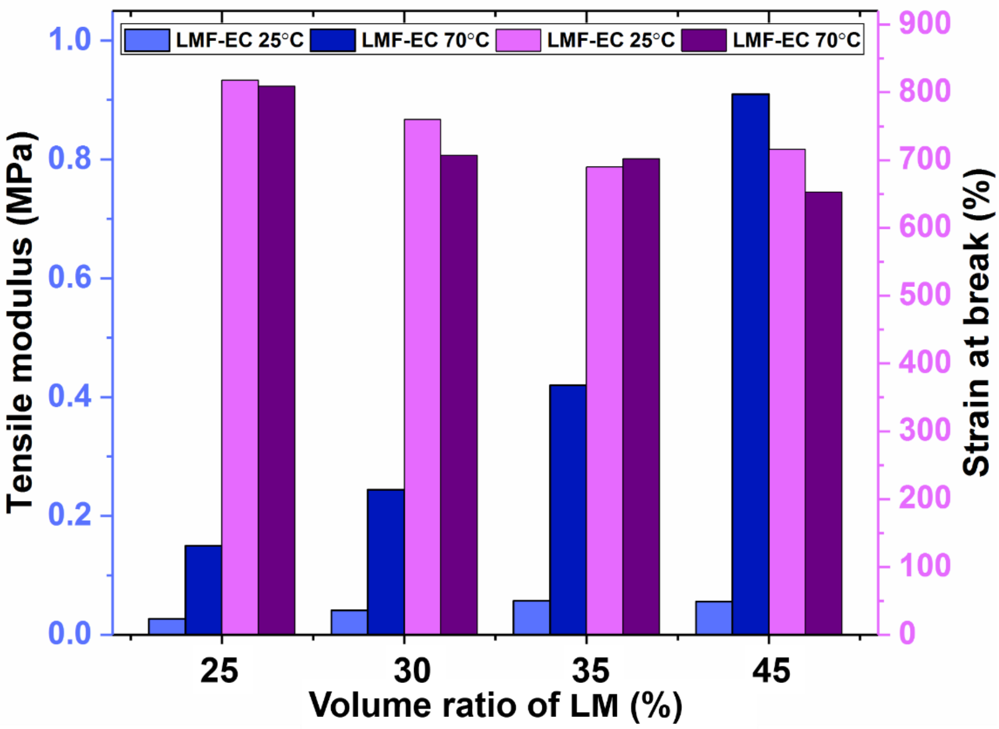

Figure S4. The tensile modulus and strains at the break of LMF-EC with different LM fractions.

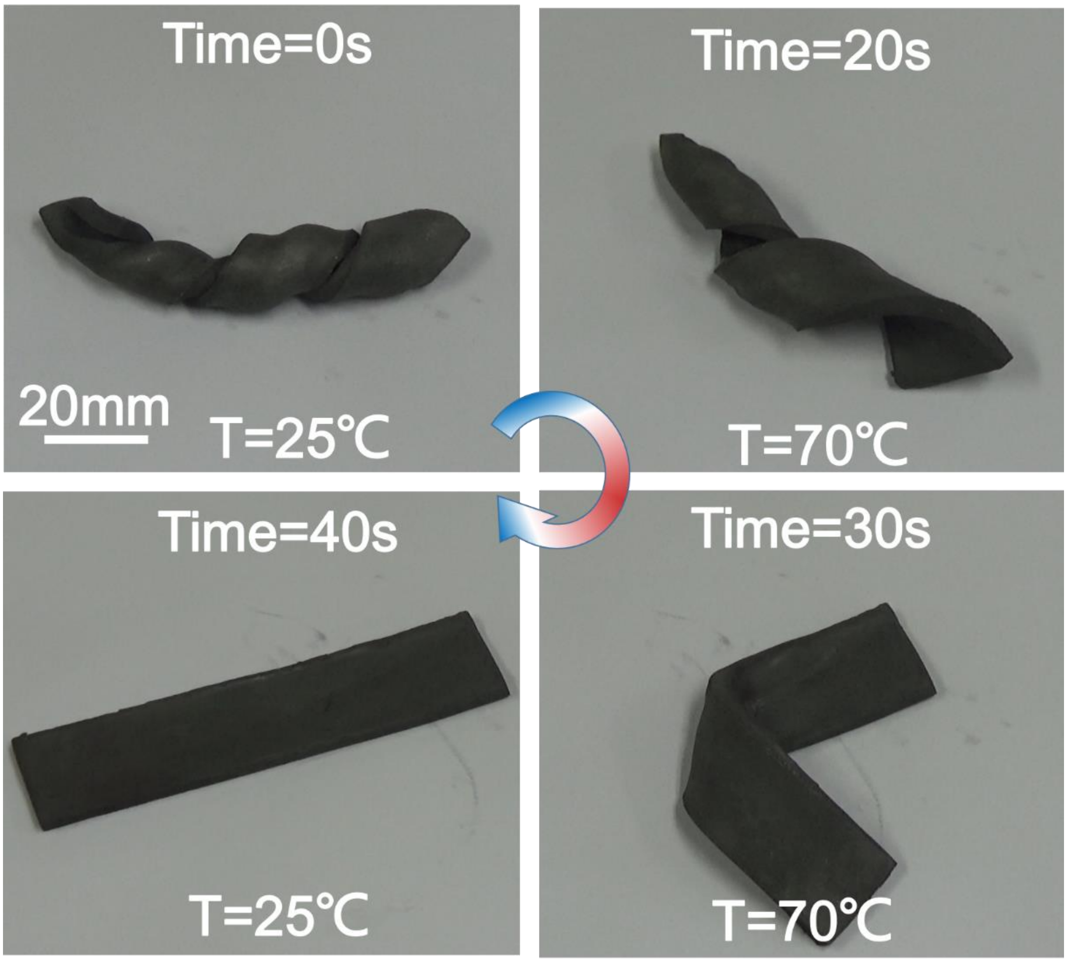

Figure S5 The thermal enabled shape-memory behaviors of LMF-EC(BiInSn) strip.

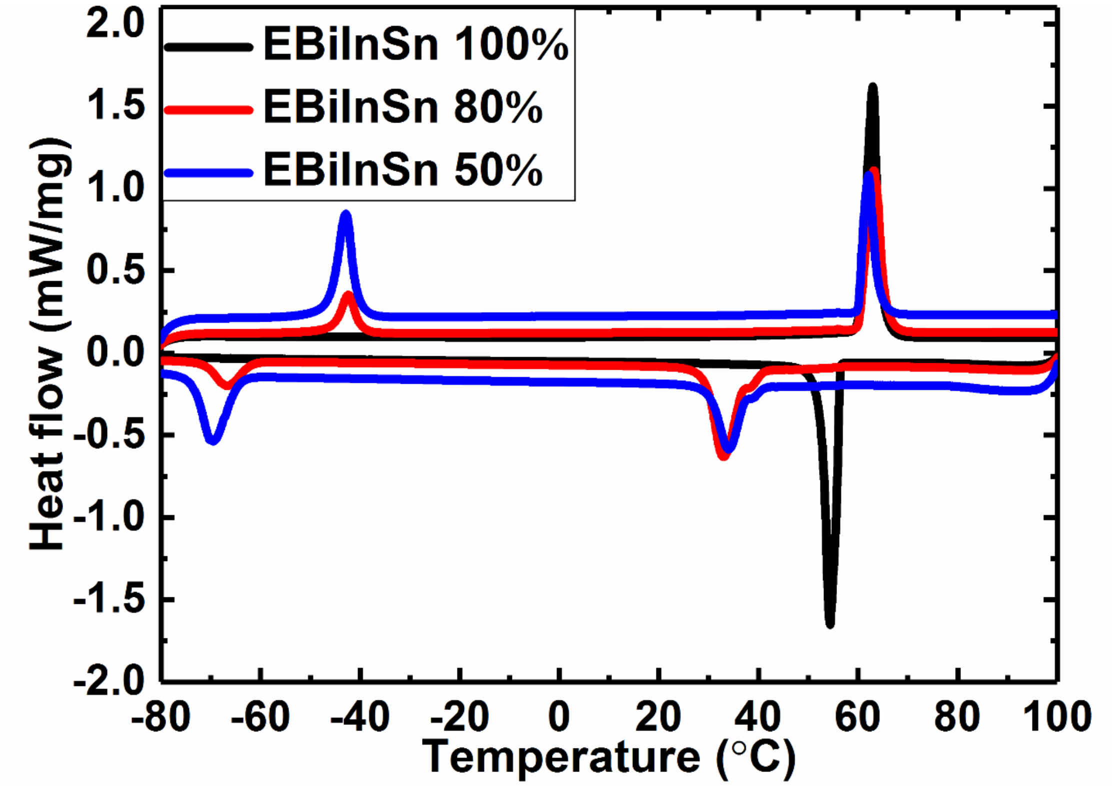

Figure S6 The DSC curves of LMEC(BiInSN).

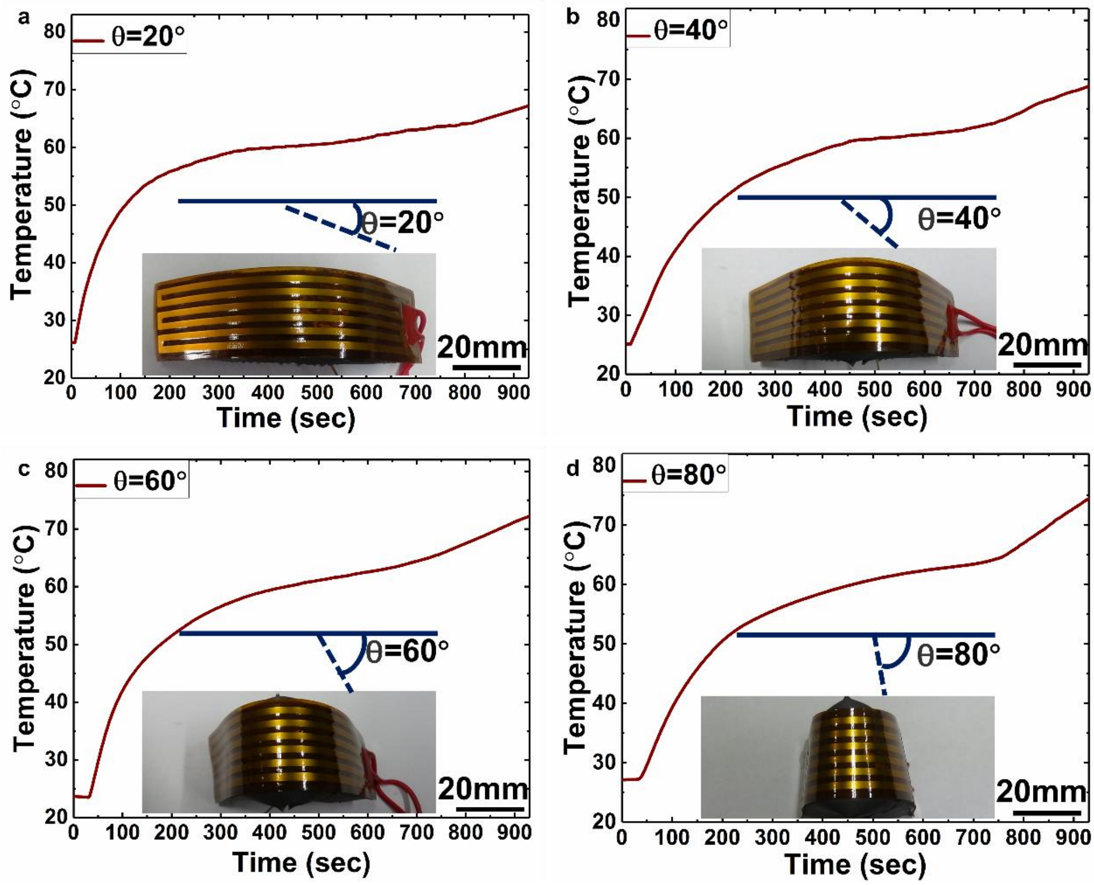

Figure S7 LMF-EC remodeled as needed to control the temperature of the flexible electronics devices with different bending angles: **a** θ=20°, **b** θ=40°, **c** θ=60°, and **d** θ=80°.

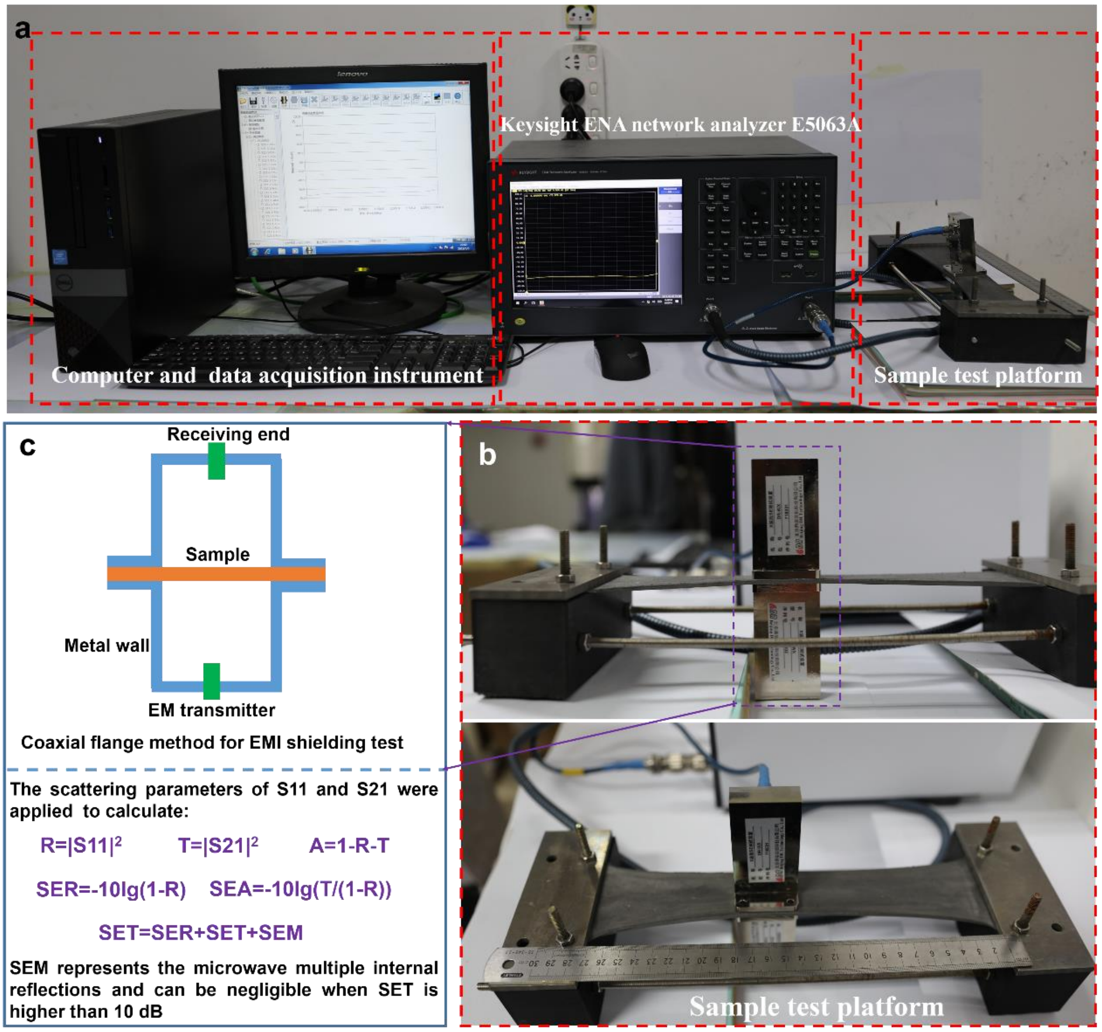

Figure S8 The test platform of EMI shielding performance. **a** The picture of test platform including data acquisition instrument, network analyzer and sample test platform. **b** The different views of the sample test platform. c The illustration of coaxial flange method for EMI shielding test, and SER, SEA and SET calculation equations.

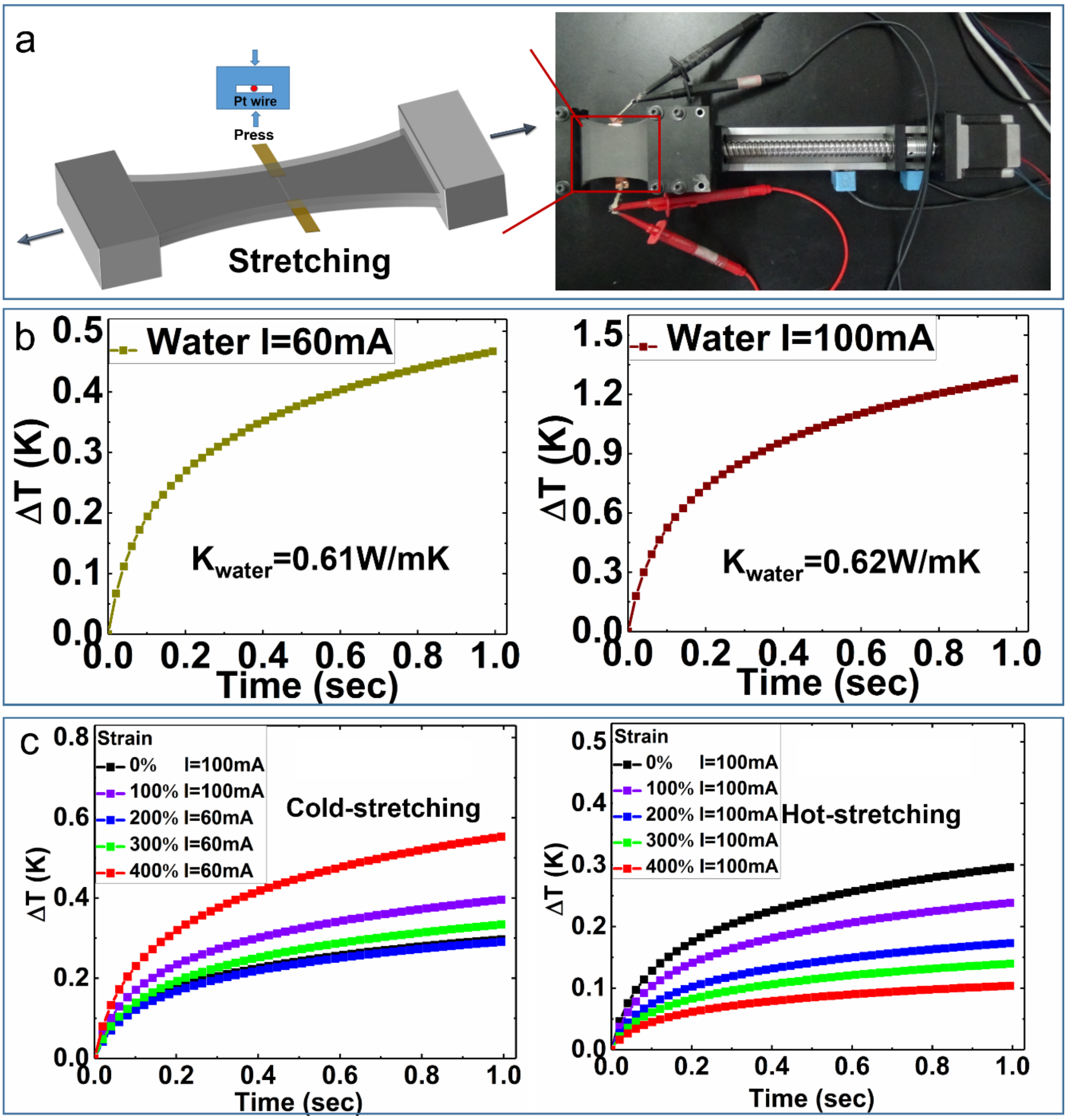

Figure S9 The test platform of LMF-EC thermal conductivity through a transient hot-wire method. **a** Illustration of Pt wire location and the photograph of the test platform. **b** Temperature rise versus time with different pulse currents and the measured thermal conductivity of water. **c** Temperature rise versus time for LMF-EC(BiInSn, ϕ =45%) with cold-stretching and hot-stretching.

Movie description:

Movie S1: 3D microstructure of LMF by micro-CT.

Movie S2: LMF-EC(Ga) has a super-stretchable capacity at room temperature.

Movie S3: The liquid conductive skeleton of LMF-EC(Ga) is considerably elongated in the stretching direction.

Movie S4: LMF-EC loaded with a 500g load in solid-state and softened and deformed under the load.

Movie S5: The thermal-mechanically enabled shape-memory function behaviors of LMF-EC.

Movie S6: The simultaneous magnetic-thermally control deformation of the flowerlike LMF-EC.

Movie S7: The stretchable LMF-EC as the electrical interconnects for LED lighting control through its thermal-mechanically enabled transition of insulator-conductor.

Movie S8: The thermal-mechanically enabled the self-healing process of LMF was observed through the microscope camera.
